# Long-term use of somatostatin analogs for chronic gastrointestinal bleeding in hereditary hemorrhagic telangiectasia

**DOI:** 10.3389/fmed.2023.1146080

**Published:** 2023-05-12

**Authors:** Raquel Torres-Iglesias, José María Mora-Luján, Adriana Iriarte, Pau Cerdà, Esther Alba, Miguel Ángel Sánchez-Corral, Ana Berrozpe, Francesc Cruellas, Enric Gamundí, Jesús Ribas, Jose Castellote, Antoni Riera-Mestre

**Affiliations:** ^1^HHT Unit, Hospital Universitari de Bellvitge, Barcelona, Spain; ^2^Internal Medicine Department, Hospital Universitari de Bellvitge, Barcelona, Spain; ^3^Bellvitge Biomedical Research Institute (IDIBELL), Barcelona, Spain; ^4^Radiology Department, Hospital Universitari de Bellvitge, Barcelona, Spain; ^5^Cardiology Department, Hospital Universitari de Bellvitge, Barcelona, Spain; ^6^Department of Digestive Diseases, Hospital Universitari de Bellvitge, Barcelona, Spain; ^7^Otorhinolaryngology Department, Hospital Universitari de Bellvitge, Barcelona, Spain; ^8^Cytology and Hematology Laboratory, Antamomic Pathology Department, Hospital Universitari de Bellvitge, Barcelona, Spain; ^9^Pneumology Department, Hospital Universitari de Bellvitge, Barcelona, Spain; ^10^Biomedical Research Networking Center on Respiratory Diseases (CIBERES), Madrid, Spain; ^11^Faculty of Medicine and Health Sciences, Universitat de Barcelona, Barcelona, Spain

**Keywords:** hereditary hemorrhagic telangectasia, gastrointestinal bleeding, anemia, somatostatin analogs, rare diseases

## Abstract

**Background:**

Chronic bleeding due to gastrointestinal (GI) involvement in patients with hemorrhagic hereditary telangiectasia (HHT) can provoke severe anemia with high red blood cells (RBC) transfusion requirements. However, the evidence about how to deal with these patients is scarce. We aimed to assess the long-term efficacy and safety of somatostatin analogs (SA) for anemia management in HHT patients with GI involvement.

**Methods:**

This is a prospective observational study including patients with HHT and GI involvement attended at a referral center. SA were considered for those patients with chronic anemia. Anemia-related variables were compared in patients receiving SA before and during treatment. Patients receiving SA were divided into responders (patients with minimal hemoglobin levels improvement >10 g/L and maintaining hemoglobin levels ≥80 g/L during treatment), and non-responders. Adverse effects during follow-up were collected.

**Results:**

Among 119 HHT patients with GI involvement, 67 (56.3%) received SA. These patients showed lower minimal hemoglobin levels (73 [60–87] vs. 99 [70.2–122.5], *p* < 0.001), and more RBC transfusion requirements (61.2% vs. 38.5%, *p* = 0.014) than patients without SA therapy. Median treatment period was 20.9 ± 15.2 months. During treatment, there was a statistically significant improvement in minimum hemoglobin levels (94.7 ± 29.8 g/L vs. 74.7 ± 19.7, *p* < 0.001) and a reduction of patients with minimal hemoglobin levels <80 g/L (39 vs. 61%, *p* = 0.007) and RBC transfusions requirement (33.9% vs. 59.3%, *p* < 0.001). Sixteen (23.9%) patients showed mild adverse effects, mostly diarrhea or abdominal pain, leading to treatment discontinuation in 12 (17.9%) patients. Fifty-nine patients were eligible for efficacy assessment and 32 (54.2%) of them were considered responders. Age was associated with non-responder patients, OR 95% CI; 1.070 (1.014–1.130), *p* = 0.015.

**Conclusion:**

SA can be considered a long-term effective and safe option for anemia management in HHT patients with GI bleeding. Older age is associated with poorer response.

## Introduction

Hereditary hemorrhagic telangiectasia (HHT) or Rendu-Osler-Weber syndrome (ORPHA774) is a rare autosomal dominant vascular disease characterized by telangiectasia and larger vascular malformations (VM) (1–3). HHT can be diagnosed either clinically using the Curaçao criteria (recurrent epistaxis, mucocutaneous telangiectasia, visceral lesions, and family history), or through molecular gene testing (4–6). Pathogenic variants in endoglin (*ENG*) and activin A receptor type II-like 1 (*ACVRL1*) genes are detected in approximately 85% of cases submitted to molecular diagnosis for clinical suspicion of HHT, causing HHT1 and HHT2, respectively (2, 7, 8). Mutations in *SMAD4* (encoding the transcription factor Smad4) have been described in less than 2% of the HHT population (9). Telangiectasis is the hallmark of HHT and consists in dilated postcapillary venules directly connected to dilated arterioles losing the normal capillary bed (10). These dilated microvessels are prone to bleeding due to fragile vessel walls and turbulent blood flow, mainly those located in nasal or gastrointestinal (GI) mucosae (2, 8, 11).

GI involvement usually becomes symptomatic by the fifth or sixth decade of life (4, 12). The prevalence of GI telangiectasia ranges from 13% to 30% in the overall HHT population to more than 90% in HHT patients with anemia (11, 13–19). GI involvement can provoke severe anemia due to chronic bleeding, with high red blood cells (RBC) transfusions and intravenous (IV) iron therapy requirements (4, 12, 15, 20, 21). However, this chronic occult bleeding is often difficult to clinically diagnose in HHT patients, as they usually also suffer from epistaxis (1, 4, 11). Recently, older age, *ENG* mutations, smoking history, and anemia have been defined as risk factors for GI involvement in HHT patients (20).

GI endoscopy techniques are recommended to detect GI telangiectasia in patients with disproportionate anemia to epistaxis severity (4). Telangiectases are usually located throughout the GI tract, limiting the effectiveness of local therapies such as argon plasma coagulation (APC). The limitation of this type of therapy usually leaves medical treatments as the only therapeutic option for patients with severe anemia despite iron replacement or RBC transfusions (4, 12, 15, 21). Tranexamic acid, bevacizumab, a humanized antibody against Vascular Endothelial Growth Factor (VEGF), and somatostatin analogs (SA) are currently the most used drugs in this scenario (22–24). Tranexamic acid is recommended for patients with mild HHT-related GI bleeding, defined as those who meet their hemoglobin goal with oral iron replacement, but not for patients who require RBC transfusions (4). SA are used subcutaneously or intramuscularly, being more comfortable for patients than IV bevacizumab. SA have been used for years in non-HHT patients with intestinal angiodysplasia and a recent meta-analysis has postulated its efficacy through improving hemoglobin levels and reducing RBC transfusions requirements (25). Although its main mechanism of action is a reduction in splanchnic blood flow, it has been hypothesized that SA could also have an anti-angiogenic effect by inhibiting VEGF (26–28). Despite SA are used in HHT patients with GI involvement, the evidence of its use is scarce and prognostic factors of long-term efficacy are unknown. We aimed to assess the efficacy and safety of SA for anemia management in HHT patients with GI involvement.

## Materials and methods

### Study design

This is an observational study on a prospective cohort including all consecutive patients attended at a referral HHT Unit in a university hospital from September 2011 to November 2022. Our Unit is the referral for patients from all over Catalonia (Spain), with a total population of seven million inhabitants. Patients with a definite HHT diagnosis according to the Curaçao Criteria (meeting ≥3 criteria) or a positive genetic test with objectively confirmed GI involvement were included in the study (4–6).

All patients provided oral consent to participate in the study according to local Clinical Research Ethics Committee requirements. We followed the Strengthening the Reporting of Observational Studies in Epidemiology (STROBE) statement guidelines for observational cohort studies (29). Personal and clinical data collected for the study are in line with the Spanish Data Protection Act (Ley Orgánica 3/2018 de 5 de diciembre de Protección de Datos Personales). The study was approved by the Clinical Research Ethics Committee of the Hospital Universitari de Bellvitge (approval number EOM029/21).

The main objective was to assess the long-term efficacy of SA for anemia management in HHT patients with GI involvement. The secondary objectives were to analyze the long-term safety of SA in the HHT scenario and to define associated risk factors for a poor response to SA.

### Clinical variables

Baseline demographic data, underlying diseases, Curaçao criteria, Epistaxis Severity Score (ESS), genetic testing, anemia-related variables, and GI involvement characteristics were collected. ESS is an online tool that quantifies epistaxis severity considering different parameters during the previous three months (30). Screening for HHT-related visceral involvement is described elsewhere (31). Anemia-related variables include: baseline hemoglobin (Hb) level at the beginning of follow-up, minimal Hb level during follow-up, severe anemia, IV iron therapy, RBC transfusion requirements, and number of RBC units transfused per patient. Minimal Hb level was selected instead of mean Hb level during follow-up because it better reflects the degree of anemia control and the severity of chronic bleeding episodes. Severe anemia was defined as minimal Hb levels <80 g/L.

GI evaluation was performed with esophagogastroduodenoscopy or colonoscopy (Olympus GIF-Q165) or endoscopic capsule (PillCamSB 3) in patients with disproportionate anemia to the amount and severity of epistaxis (4). Telangiectasias were classified according to their number (few: ≤ 10 telangiectasias or multiple: >10 telangiectasias) and size (small: ≤ 3 mm or large: >3 mm) (13). Previous use of APC therapy before SA treatment was also collected.

### SA therapy groups

Not all patients with HHT and GI involvement were considered for SA treatment. At our HHT Unit, patients were considered for SA if they presented chronic anemia (Hb < 120 g/L for females and Hb <130 g/L for males) disproportionate to epistaxis despite IV iron or RBC transfusion requirements, or severe anemia. Patients who met these criteria received information on the use of SA, including potential risks and benefits. Given the study’s observational nature, conducted within clinical practice, there was no specific protocol for the initial treatment dose. Therefore, starting doses could vary between daily subcutaneous doses of octreotide (50 mcg bid, 100 mcg bid, or 100 mcg tid), monthly intramuscular doses of octreotide long-acting release (LAR) (10 mg, 20 mg, or 30 mg), or lanreotide (60 or 120 mg). For maintenance treatment, a switch to monthly dosing was attempted for convenience. All patients were prospectively followed-up and SA doses were titrated according to tolerance and Hb levels during follow-up. Side effects during this follow-up period were collected.

For efficacy analysis, those patients who received less than 2 months of daily doses or less than three monthly doses of SA were excluded. SA patients were divided into two subgroups according to treatment response. Responder patients were defined as those who showed minimal Hb levels improvement >10 g/L during SA therapy without severe anemia (minimal Hb levels <80 g/L). Non-responder patients were defined as those with no or less than 10 g/L minimal Hb levels improvement or those who persist with severe anemia.

### Statistical analysis

A descriptive statistical analysis was performed for categorical and continuous variables and expressed as proportions or means with standard deviations (SD), respectively. Baseline clinical characteristics and long-term outcomes were compared between patients with and without SA therapy. Categorical variables were compared with the Chi-square test or the Fisher exact test, whereas the t-test was used to compare continuous variables. The Kolmogorov–Smirnov test was performed for continuous variables to assess normality. Non-parametric variables were expressed as median (interquartile range -IQR-) and compared with the U Mann–Whitney test.

For safety evaluation, the number and type of adverse effects and the need for SA treatment withdrawal were assessed. For efficacy assessment, anemia-related variables were compared before and during SA treatment, using the *t*-test for related parametric samples and Wilcoxon signed-rank test for non-parametric related samples. Related qualitative samples were studied with McNemar’s test.

Logistic regression analyses were performed to identify associated risk factors for non-response to SA treatment. The selection of variables included in the multivariate model was based on clinical and statistical significance, using variables with value of *p* <0.1 on the univariate analysis, and adjusting the number of variables used to the sample size. Missing data were not imputed for the multivariate analyses. Odds ratio (OR) and 95% confidence interval (CI) were used to quantify the association. A value of *p* of <0.05 was considered statistically significant. Analyses were performed using IBM SPSS Statistics, version 22.0 for the PC (IBM Corp., Armonk, NY, USA).

## Results

### Clinical characteristics

During the study period, 430 patients were attended at our HHT Unit. Amongst these, 119 patients had GI involvement objectively confirmed by GI endoscopy techniques. Most patients (66; 55.5%) were female and the mean age was 57.66 ± 12.53 years. Clinical diagnosis of HHT was definite according to the Curaçao Criteria (meeting ≥3 criteria) in all patients. A genetic test was carried out in 110 (92.4%) patients: 53 (44.5%) had *ENG* mutations and 51 (42.8%) had *ACVRL1* mutations, and no pathogenic variants were found in 6 (5%) patients.

SA was initiated in 67 patients. These patients had higher ESS, lower baseline Hb levels at the first visit, lower minimal Hb levels during follow-up, and more RBC transfusions and IV iron therapy requirements than non-SA patients. No statistically significant differences were found in underlying diseases, Curaçao criteria, or genetic testing results between both groups. Regarding GI involvement, SA patients had more frequently multiple (>10) and larger (>3 mm) telangiectases, jejunum involvement, and APC therapy requirements than non-SA patients (Table 1).

**Table 1:** Clinical characteristics comparing SA and non-SA patients.

	SA patients (*n* = 67)	Non-SA patients (*n* = 52)	*p*-value
Demographic characteristics			
Gender (female), *n* (%)	34 (50.7)	32 (61.5)	0.240
Age years-old, mean (SD)	58.2 (±11.5)	56.9 (±13.8)	0.557
Underlying diseases, *n* (%)			
Smoking history	43 (64.2)	26 (50)	0.120
Alcoholism	10 (14.9)	11 (21.2)	0.377
Hypertension	28 (41.8)	20 (38.5)	0.713
Diabetes Mellitus	13 (19.4)	8 (15.4)	0.568
Dyslipemia	19 (28.4)	21 (40.4)	0.168
Chronic heart disease	18 (26.9)	8 (15.4)	0.133
Chronic lung disease	16 (23.9)	11 (21.2)	0.725
Atrial fibrillation	15 (22.4)	6 (11.5)	0.124
Curaçao criteria			
Epistaxis, *n* (%)	67 (100)	52 (100)	
ESS at first visit, mean (SD)	4.75 (±1.91)	3.42 (±1.67)	<0.001
Family history, *n* (%)	64 (95.5)	49 (94.2)	0.653
Mucocutaneous telangiectases, *n* (%)	67 (100)	52 (100)	
Visceral involvement, *n* (%)	67 (100)	52 (100)	
Genetic test, *n* (%)			
*ENG*	31 (46.3)	22 (42.3)	0.950
*ACVRL1*	31 (46.3)	20 (38.5)	0.607
Negative	2 (3)	4 (7.7)	0.234
Anemia related variables			
Hemoglobin levels at first visit (g/L), mean (SD)	111.5 (±27.0)	121.8 (±27.3)	0.030
Minimal hemoglobin levels (g/L), median [IQR]	73 [60–87]	99 [79.2–122.5]	<0.001
IV iron therapy	57 (85.1)	24 (46.2)	0.001
RBC transfusion, *n* (%)	41 (61.2)	20 (38.5)	0.014
Number RBC transfusions, median [IQR]	3 [0–15]	3 [0–3]	0.012
Gastrointestinal involvement			
Multiple telangiectases (>10), *n* (%)	41 (61.2)	18 (36.7)	0.005
Size > 3 mm, *n* (%)	34 (50.7)	11 (22.4)	0.001
APC therapy, *n* (%)	32 (47.8)	11 (21.2)	0.004
Site of GI telangiectases, *n* (%; total *n*)			
Gastric	46 (68.7; 67)	33 (64.7; 51)	0.651
Duodenal	40 (60.6; 66)	32 (62.7; 51)	0.814
Jejunal	55 (96.5; 57)	28 (80; 35)	0.024
Ileal	35 (62.5; 56)	19 (55.9; 34)	0.534
Colonic	18 (45; 40)	11 (37.9; 29)	0.557

### Efficacy and safety of SA patients

Initial doses ranged from daily doses of octreotide in nine (13.4%) patients, monthly doses of octreotide LAR in 51 (76.1%), and monthly doses of lanreotide in seven (10.4%) patients. Most patients (94.9%) followed maintenance treatment with monthly doses of octreotide LAR (51 patients) or lanreotide (5 patients). During the treatment period (20.9 ± 15.2 months), 16 (23.9%) patients experienced side effects and seven of them were receiving daily doses of octreotide. These events were diarrhea or abdominal pain in all cases and one patient also showed a biliary colic episode. Side effects led to SA withdrawal in 12 (17.9%) patients.

**Figure 1:**
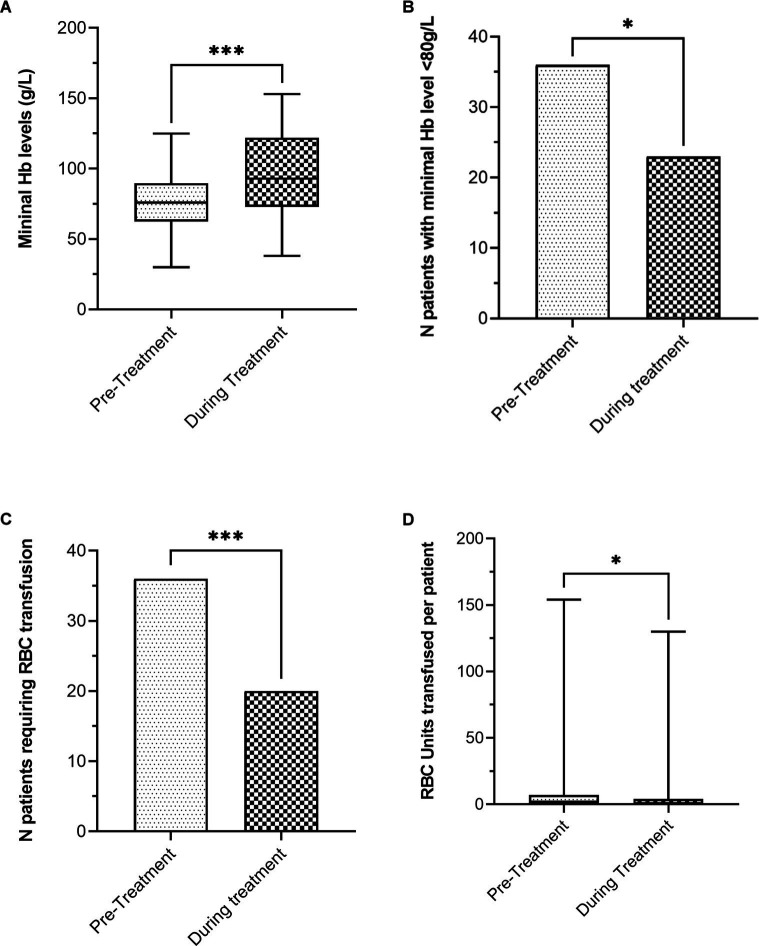
Efficacy outcomes before and during treatment with SA. **(A)** Mean minimal hemoglobin levels (g/L). **(B)** Number of patients with minimal hemoglobin level < 80 g/L. **(C)** Number of patients requiring RBC transfusion. **(D)** RBC units transfused per patient (*p < 0.05; ***p < 0.001).

For the efficacy assessment, eight patients were excluded. Six of them because they received less than 2 months of daily doses or less than three monthly doses of SA due to side effects, and the other two because no enough available data. Thus, 59 patients were analyzed for efficacy. During treatment with SA, patients showed higher mean minimum Hb levels (94.7 ± 29.8 g/L vs. 74.7 ± 19.7 g/L, *p* < 0.001) and fewer patients showed persistent severe anemia (39% vs. 61%, *p* = 0.007) than before SA therapy. Moreover, once treatment was started, fewer patients needed transfusions (33.9% vs. 59.3%, p < 0.001) and patients needed a lower median of RBC units per patient (0 [0–4] vs. 2 [0–7], *p* = 0.006) (Table 2; Figure 1).

**Table 2:** Efficacy outcomes in SA patients (*n* = 59) before and during treatment.

	Before SA	During SA	*p*-value
Minimal hemoglobin levels (g/L), mean (SD)	74.7 (±19.7)	94.7 (±29.8)	<0.001
Severe anemia (minimal Hemoglobin levels < 80 g/L), *n* (%)	36 (61)	23 (39)	0.007
N of patients requiring RBC transfusion	35 (59.3)	20 (33.9)	<0.001
RBC units per patient, median [IQR]	2 [0–7]	0 [0–4]	0.006

### Clinical characteristics of patients according to SA response and risk factors for non-response

Despite the aforementioned improvement of anemia-related variables during SA therapy, there were 27 (45.8%) patients considered non-responders. Thus, the real efficacy of SA for the management of chronic anemia in HHT patients with GI chronic bleeding was 54.2%. Non-responder patients were older (61.1 ± 9.2 vs. 53.6 ± 11.8, *p* = 0.010), with a higher prevalence of hypertension (55.6% vs. 28.1%, *p* = 0.033), and previous use of APC therapy (66.7% vs. 31.3%, *p* = 0.007) than responder patients. Before SA treatment, non-responder patients showed a lower mean of minimal Hb levels (69.1 ± 23 g/L vs. 79.44 ± 15.3 g/L, *p* = 0.044) and higher RBC transfusion requirements (81.5% vs. 43.8%, *p* = 0.003) than responder patients. During treatment period, mortality was higher in non-responder patients than responder patients (14.8% vs. 0%, *p* = 0.039). Although no statistically significant differences were observed, there is a tendency to have more ileal involvement in responder patients (45.5% vs. 71.4%, *p* = 0.063). No significant differences in any other underlying diseases, genetics, ESS, or characteristics of GI involvement were detected between both groups (Table 3).

**Table 3:** Clinical characteristics and outcomes according to response to SA therapy.

	Responder patients (*n* = 32)	Non-responder patients (*n* = 27)	*p*-value
Treatment period (months), mean (SD)	22.32 (±15.5)	19.83 (±15.0)	0.537
Clinical characteristics			
Gender (female), *n* (%)	19 (59.4)	11 (40.7)	0.154
Age years-old, mean (SD)	53.6 (±11.8)	61.1 (±9.2)	0.010
Underlying diseases, *n* (%)			
Smoking history	23 (71.9)	18 (66.7)	0.665
Alcoholism	5 (15.6)	4 (14.8)	1
Hypertension	9 (28.1)	15 (55.6)	0.033
Diabetes Mellitus	4 (12.5)	8 (29.6)	0.103
Dyslipemia	9 (28.1)	7 (25.9)	0.850
Chronic heart disease	8 (25)	10 (37)	0.317
Chronic lung disease	6 (18.8)	8 (29.6)	0.328
Atrial fibrillation	7 (21.9)	8 (29.6)	0.496
Curaçao Criteria			
Epistaxis, *n* (%)	32 (100)	27 (100)	
ESS, mean (SD)	4.47 (±1.83)	5.16 (±1.49)	0.117
Family history, *n* (%)	31 (96.9)	26 (96.3)	1
Mucocutaneous telangiectasia, *n* (%)	32 (100)	27 (100)	
Visceral involvement, *n* (%)	32 (100)	27 (100)	
Genetic test, *n* (%)			
ENG	16 (50)	9 (33.3)	0.243
ACVRL1	15 (46.9)	15 (55.6)	0.386
Gastrointestinal involvement			
Multiple telangiectases (>10), *n* (%)	14 (51.8)	15 (55.6)	0.390
n telangiectases, mean (SD)	12 (±2)	96 (±76)	0.389
Size > 3 mm, *n* (%)	14 (43.7)	18 (66.7)	0.129
APC therapy, *n* (%)	10 (31.3)	18 (66.7)	0.007
Site of GI telangiectases, *n* (%; total *n*)			
Gastric	19 (59.4)	21 (77.8)	0.132
Duodenal	16 (50)	17 (63)	0.239
Jejunal	28 (100; 28)	20 (90.9; 22)	0.189
Ileal	20 (71.4; 28)	10 (45.5; 22)	0.063
Colonic	6 (42.9; 14)	10 (50; 20)	0.681
Anemia related variables before SA			
Hemoglobin levels at first visit (g/L), mean (SD)	113.8 (±23.3)	107.2 (±21.9)	0.266
Minimal hemoglobin levels (g/L), mean (SD)	79.4 (±15.3)	69.1 (±23)	0.044
Severe anemia (minimal hemoglobin levels < 80 g/L), *n* (%)	17 (53.1)	19 (70.4)	0.176
IV iron therapy	27 (84.4)	24 (88.9)	0.715
RBC transfusion, *n* (%)	14 (43.8)	22 (81.5)	0.003
RBC units per patient, median [IQR]	0 [0–2]	7 [2–31]	0.004
Mortality, *n* (%)	0 (0)	4 (14.8)	0.039

After multivariate analysis, only age was associated with non-responder patients (OR 1.070, 95% CI 1.014–1.130; *p* = 0.015).

## Discussion

Our study provides the longest follow-up of HHT patients with GI involvement treated with SA and supports that this therapy is an effective treatment for the management of anemia due to chronic GI bleeding in HHT patients. In fact, according to our results, patients showed a statistically significant increase of 20 g/L in the minimum Hb levels and lower RBC transfusion requirements during SA treatment. These results are in line with other studies using SA for GI bleeding in HHT and mostly in non-HHT patients (25–28, 31). Kroon et al. (28), in a non-randomized clinical trial including 11 HHT patients, showed a reduction in RBC units transfused from 13.5 to 8 units during 6 months of follow-up. Holleran et al. (26), in an open-label, uncontrolled proof-of-concept study including 24 non-HHT patients with small bowel angiodysplasias treated with octreotide LAR during 6 months, showed an increase of 20 g/L in mean Hb levels and a 70% reduction in the number of patients who required RBC transfusions. A recent meta-analysis of 11 studies including 212 non-HHT patients with GI angiodysplasias, support the effectiveness of SA with an improvement of Hb levels by 30 g/L and a reduction of more than 50% of RBC transfusion requirements in 83% of patients (25). In this meta-analysis, low doses of LAR formulation were equally effective as high doses and better tolerated, whereas in our study, this analysis could not be performed due to the small sample size. In our study, after a longer treatment period, 54.2% of patients who received SA showed an improvement >10 g/L in the minimum Hb levels and remained without severe anemia. It is important to point out that patients who received SA had more severe GI involvement than non-SA patients and, consequently, the possible benefit of SA therapy in the early stages needs further investigation.

Though not clinically relevant, SA patients also showed statistically significant higher ESS at first visit than non-SA patients. This relationship could be explained by a more severe microvessel-predominant pattern, as telangiectasis is the pathological hallmark in both nasal and GI mucosae. In fact, it has been described a higher ESS in HHT patients with GI involvement and severe anemia or transfusion requirements (20). Many cases of anemia are misattributed to overt epistaxis instead of attributing to GI bleeding among patients with HHT (32). Because both types of bleeding can coexist, a high clinical suspicion of GI bleeding in patients with severe anemia is necessary despite a high ESS.

In addition to age, non-responder patients had a more frequent history of hypertension and previous use of APC. Hypertension is a known risk factor for epistaxis that should be actively treated in HHT patients (33). Previous use of APC may reflect more severe GI involvement before initiating treatment with SA. Even though these findings, after multivariate analysis, only age was associated with a poor response to SA treatment. This finding could be related to more severe GI involvement in these patients since GI is associated with age in HHT patients (4, 12). We did not find similar results described in non-HHT patients (25, 34).

Regarding safety, 16 (23.9%) patients showed side effects and SA had to be withdrawn in 12 (17.9%) of them. All side effects were mild and related to diarrhea or abdominal pain. Interestingly, seven out of nine patients receiving daily doses of SA developed side effects. Thus, monthly formulation seems to be better tolerated than the daily dose of octreotide and showed no relevant side effects. This observation is in accordance with two previous studies in non-HHT patients, one including 11 patients with severe GI bleeding using LAR 20 mg dose after a median follow-up of 15 (IQR 5–48) months and the other one including 27 patients with GI angiodysplasias and obscure GI bleeding using lanreotide for at least 6 months (35, 36). Because anti-angiogenic drugs for GI involvement in HHT, such as oral thalidomide, often show serious adverse effects, and there are no data on the long-term safety of IV bevacizumab, subcutaneous or intramuscular SA can be considered an option both in terms of efficacy and safety (37).

There are some limitations and strengths of our study that should be mentioned. First, it is a single-center observational study without a control group. However, our study represents the largest published series of HHT patients treated with SA, reflecting the wide spectrum of HHT patients with GI involvement. Second, the lack of a protocolized dose made it difficult to assess the optimal doses of SA for these patients. Third, anemia-related variables are collected from the beginning of follow-up, and not in a limited period of time before SA therapy. However, we consider it appropriate to show the evolution during the entire follow-up, both before and during SA treatment period, because a shorter observation period may not reflect the fluctuating bleeding course of HHT patients and may bias bleeding episodes severity. Moreover, the broad long-term follow-up reinforces the robustness of our results and allows for a better assessment of outcomes. Fourth, difficulties in attributing low Hb levels to epistaxis or GI bleeding could be another inherent limitation. Finally, the definition of “improvement in minimal Hb level” is arbitrary and may be clinically debatable. However, we aimed to provide objective and useful results for clinicians dealing with this complex manifestation with such scant evidence.

In conclusion, SA can be considered an effective long-term therapy for HHT patients with GI bleeding and chronic anemia. Side effects were mild diarrhea or abdominal pain, mostly with daily doses of octreotide, and led to SA withdrawal in less than one in every five patients. Age was associated with a poor response to SA treatment.

## Data availability statement

The raw data supporting the conclusions of this article will be made available by the authors, without undue reservation.

## Ethics statement

The studies involving human participants were reviewed and approved by EOM029/21. Written informed consent for participation was not required for this study in accordance with the national legislation and the institutional requirements.

## Author contributions

RT-I, JM-L, and AR-M: study concept and design. RT-I, JM-L, PC, AI, EA, AB, and EG: acquisition of data. RT-I, PC, JR and AR-M: statistical analysis. RT-I, JM-L, MS-C, FC, EG, JR, JC, and AR-M: analysis and interpretation of data. RT-I, JM-L, PC, and AR-M: drafting of the manuscript. JR, JC, and AR-M: critical revision of the manuscript. PC and AR-M: obtained grant support. All authors contributed to the article and approved the submitted version.

## Funding

This study has been funded by Instituto de Salud Carlos III through the projects PI20/00592 and FI21/00007, co-funded by European Regional Development Fund (ERDF), “a way to build Europe.”

## Conflict of interest

The authors declare that the research was conducted in the absence of any commercial or financial relationships that could be construed as a potential conflict of interest.

## Publisher’s note

All claims expressed in this article are solely those of the authors and do not necessarily represent those of their affiliated organizations, or those of the publisher, the editors and the reviewers. Any product that may be evaluated in this article, or claim that may be made by its manufacturer, is not guaranteed or endorsed by the publisher.

## Abbreviations

*ACVRL1*: activin A receptor type II-like 1 gene
APC: argon plasma coagulation
CI: confidence interval
*ENG*: endoglin gene
ESS: epistaxis severity score
GI: gastrointestinal
Hb: hemoglobin
HHT: hereditary hemorrhagic telangiectasia
IQR: interquartile range
IV: intravenous
LAR: long-acting release
OR: odds ratio
RBC: red blood cell
SA: somatostatin analogs
SD: standard deviation
VEGF: Vascular Endothelial Growth Factor
VM: vascular malformation
